# Fatal Subarachnoid Hemorrhage in a Deep Brain Stimulation Patient: Displacement of Stimulation Leads for Deep Brain Stimulation Indicate Subarachnoid Hemorrhage on X-ray

**DOI:** 10.3390/diagnostics14020222

**Published:** 2024-01-19

**Authors:** Gregor Bara, Valeri Borger, Jaroslaw Maciaczyk

**Affiliations:** Department of Neurosurgery, University Hospital Bonn, Venusberg-Campus 1, Building 81, 53127 Bonn, Germany

**Keywords:** deep brain stimulation, subarachnoid hemorrhage, intracerebral hemorrhage

## Abstract

We depict the rare case of a patient with aneurysmatic subarachnoid hemorrhage previously treated with deep brain stimulation for Parkinson’s disease. Initial CT scans showed a Fisher grade 4 subarachnoid hemorrhage with lead displacement due to midline-shift. CT angiogram revealed a supra-ophthalmic aneurysm of the internal carotid artery. The patient subsequently underwent clipping of the aneurysm and decompressive hemicraniecomy.

A 70-year-old patient was admitted to our emergency room with a history of sudden loss of consciousness and epileptic seizure. An initial GCS (Glasgow Coma Scale) was 3, and pupils were isochoric with intact direct and consensual light reflex. Sixteen years prior, he had been implanted with bilateral stimulation leads for deep brain stimulation of the subthalamic nucleus due to Parkinson’s disease. An initial head CT scan (Philips Icon, 1mm head scan) showed a predominantly left-sided subarachoid hemorrhage (SAH) Fisher grade 4 with intra-ventricular hemorrhage, intra-parenchymal hemorrhage within the left frontal lobe, subdural hematoma, and midline-shift of 12 mm. The SAH was classified as WFNS (World Federation of Neurological Surgeons) grade 5. The CT angiogram (Philips Icon, 1mm head scan) of the supra-aortal vessels revealed a left-sided supra-ophthalmic aneurysm of the internal carotid artery as the supposed source of hemorrhage. An external ventricular drain was placed and the patient subsequently brought to the operating room for clipping of the aneurysm and decompressive hemicraniectomy. During the latter, the left-sided DBS stimulation lead had to be removed. Eventually, the therapy was switched to best supportive care due to massive parenchyma destruction.

A subarachnoid hemorrhage (SAH) is an extravasation of blood into the space between the arachnoid membrane and the pia mater filled with cerebrospinal fluid. The most common cause of a non-traumatic SAH is due to a ruptured intracranial aneurysm [1].

Whilst aneurysmal SAH can be a devastating condition associated with neurological complications up to death, nonaneurysmal SAH is associated with a good prognoses and is rarely associated with neurological complications [2].

Diagnosis of a SAH is primarily performed by computed tomography (CT) of the head, in which the SAH appears characteristically with hyperdense extravasated blood sometimes associated with intraparenchymal hematoma and hydrocephalus [3,4].

In this case, however, first signs of the SAH could be identified in the initial head X-ray of the CT scout image due to the patient having been implanted with bilateral stimulation leads for deep brain stimulation (Figure 1). The differential diagnosis of an intracranial mass affect includes pathologies of traumatic (epidural hematoma, acute subdural hematoma, chronic subdural hematoma, traumatic intracerebral hemorrhage), tumorous (brain derived tumors such as glioblastoma multiforma or meningioma, metastases) as well as vascular origin (subarachnoid hemorrhage, intracranial hemorrhage). Table 1 depicts the most common differential diagnoses in respect to pathological origin, entity, CT morphology and clinical dynamic. In this particular case, the CT scan revealed the brain shift to be due to the subarachnoid hemorrhage with an associated hematoma which displaced the stimulation leads to the contralateral side (Figure 2). The CT angiogram showed a supra-ophthalmic aneurysm of the internal carotid artery to be the source of hemorrhage (Figure 3). 

The amount of blood within the subarachnoid space and the ventricles, as well as the parenchyma, can be easily graded on a head CT scan and is correlated with poor outcome [5,6]. Further, the prognosis is correlated to the initial clinical exam which is most widely measured on the clinical scale of Hunt and Hess and the World Federation of Neurological Surgeons [7,8]. Given the clinical and radiological presentation of this patient, this was defined as poor grade. 

Once an aneurysm has been identified by CT angiogram and/or digital subtraction angiography, treatment can be performed either with endovascular coiling or microsurgical clipping [9].

Further complications can be managed by placement of an external ventricular drain for hydrocephalus and decompressive hemicraniectomy in case of otherwise uncontrollable intracranial hypertension, as performed in this case report (Figure 4) [10].

## Figures and Tables

**Figure 1:**
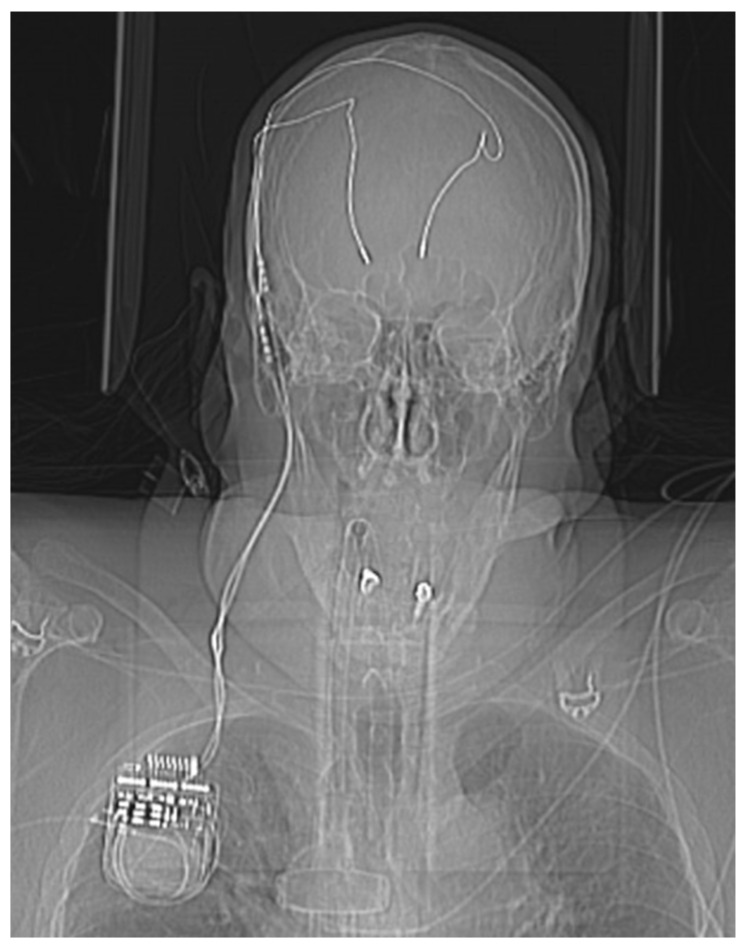
Initial scout image of the head CT scan, essentially mimicking an AP cranial X-ray. Note the implanted stimulations leads for deep brain stimulation, which are connected to an infra-clavicular neurostimulator. The DBS stimulation leads are displaced to the right side.

**Figure 2:**
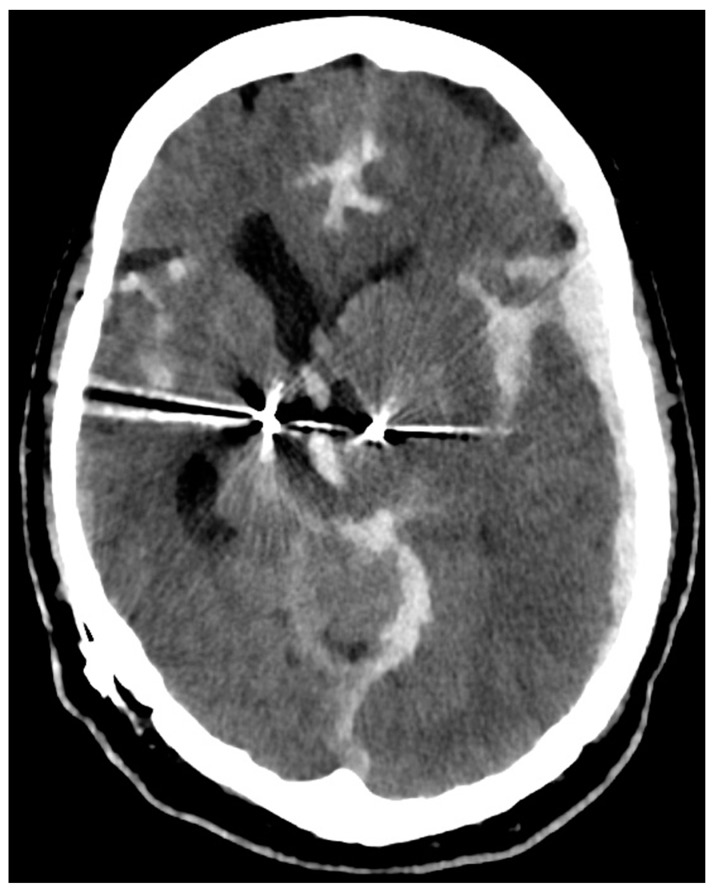
Initial head CT scan shows the predominantly left-sided subarachnoid hemorrhage with intra-ventricular hemorrhage, subdural hematoma, and midline-shift. The DBS stimulation leads are displaced to the contralateral side.

**Figure 3:**
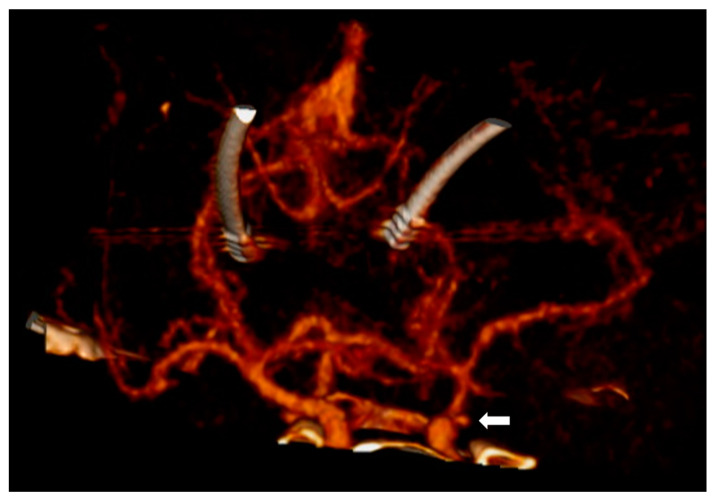
A 3D reconstruction of the CT angiogram showing a supra-ophthalmic aneurysm of the internal carotid artery as the source of hemorrhage (marked by the arrow). DBS leads are displaced to the contralateral side.

**Figure 4:**
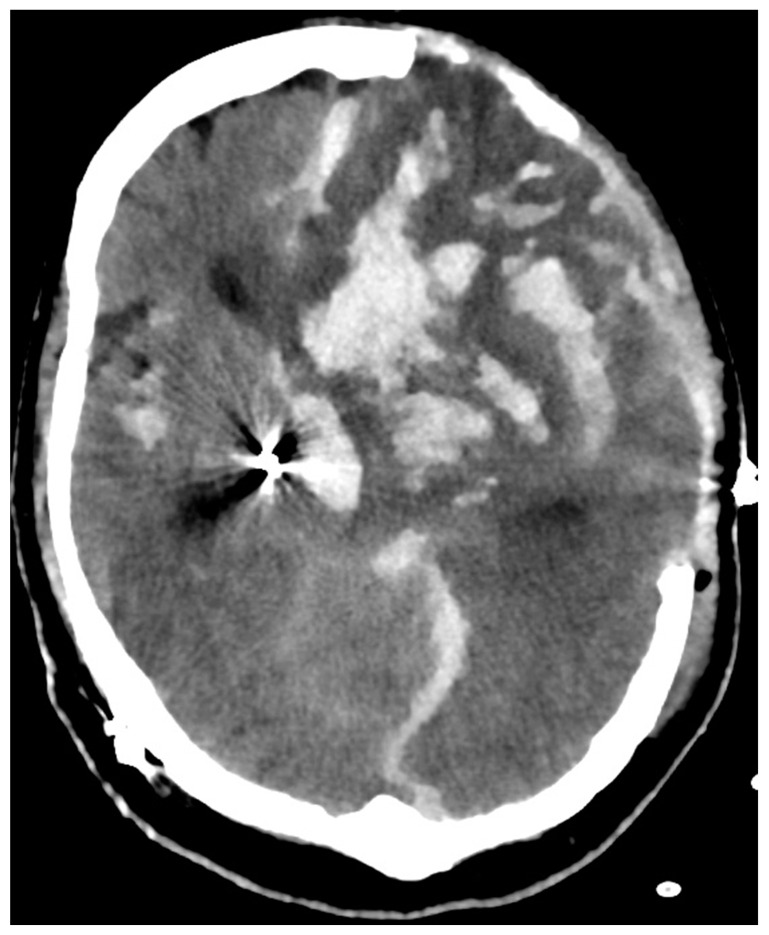
Postoperative head CT scan after clipping of the supra-ophthalmic aneurysm, left-sided DBS lead removal and decompressive hemicraniectomy. Eventually, therapy was switched to best supportive care due to massive parenchyma destruction.

**Table 1:** Differential diagnosis of mass effect.

Pathological Class	Pathology	CT Morphology	Clinical Dynamic
Traumatic	Epidural hematoma	Hyperdense	Minutes to hours
		biconvex	
		extra-axial	
	Acute subdural hematoma	Hyperdense	Minutes to hours
		crescent-shaped	
		extra-axial	
		spreading diffusely over the affected hemisphere	
	Chronic subdural hematoma	Hypodense	Weeks
		crescent-shaped	
		extra-axial	
		spreading diffusely over the affected hemisphere	
		septation and sediment effect	
	Traumatic intracerebral hemorrhage	Hyperdense	Minutes to hours
		Intra-axial	
		Usually in combination with traumatic subdural hematoma and/or acute subdural hematoma	
Tumorous	Brain derived such as glioblastoma multiforme, meningeoma	GBM: intra-axial mass with thick, irregularly enhancing margins with a central necrotic core, possibly hemorrhagic	Weeks to months
		Meningeoma: extra-axial mass, well-circumscribed, contract to dura	
	Metastasis	Intra-axial	Weeks to months
		Subcortical	
		Usually multiple	
Vascular	Subarachnoid hemorrhage	Hyperdense	Sudden onset
		Usually extra-axial within the subarachnoid space and cisterns, but intracerebral hemorrhage may occur	
		Hydrocephalus may occur	
	Intracerebral hemorrhage	Hyperdense	Sudden onset
		Intra-axial	
		Usually thalamus, caudate nucleus and pons	
		Hydrocephalus may occur	

## Data Availability

No new data was created.
